# Carbon stocks of particle board and fiberboard in Japan

**DOI:** 10.1038/s41598-023-37132-x

**Published:** 2023-06-17

**Authors:** Chihiro Kayo, Kotoko Sanjo, Issei Sato, Mengyuan Liu, Gianova Vierry Prasetyadi, Suguru Hirahara

**Affiliations:** 1 Institute of Agriculture, Tokyo University of Agriculture and Technology, 3-5-8 Saiwai-Cho, Fuchu, Tokyo 183-8509 Japan; 2 United Graduate School of Agricultural Science, Tokyo University of Agriculture and Technology, 3-5-8 Saiwai-Cho, Fuchu, Tokyo 183-8509 Japan; 3 Faculty of Forestry, Universitas Gadjah Mada, Bulaksumur, Yogyakarta, 55281 Indonesia

**Keywords:** Climate sciences, Environmental sciences, Environmental social sciences

## Abstract

The carbon stock function of harvested wood products (HWPs) is attracting attention among climate change countermeasures. Among HWPs, particle board (PB) and fiberboard (FB) mainly use recycled materials. This study estimated carbon stocks of PB and FB and their annual changes over the past 70 years in Japan using three methods of the Intergovernmental Panel on Climate Change guidelines: Tiers 1–3. Tier 1 uses first order decay (FOD), a 25-year half-life, and the Food and Agriculture Organization of the United Nations database. Tier 2 uses FOD, a 25-year half-life, and Japan-specific statistics. Tier 3 uses a log-normal distribution for the decay function and a 38–63-year half-life of building PB/FB. Japan’s PB and FB carbon stocks have increased for the past 70 years. The latest carbon stock in early 2022 and the annual change in carbon stock in 2021 was 21.83 million t-C and 0.42 million t-C/year, respectively for Tier 3. Tier 3 has the highest estimation accuracy by using decay functions and half-lives that match the actual conditions of building PB and FB, whereas Tiers 1 and 2 were underestimates. Approximately 40% of the carbon stock is derived from waste wood, which extends its utilization.

## Introduction

The Paris Agreement was adopted in 2015 with the aim of resolving climate change, one of the most serious environmental problems facing humankind. The agreement has the following common global long-term goals: ensure that the increase in global average temperature is well below 2 °C from that of pre-industrial levels, pursue efforts to limit it to 1.5 °C, and achieve a balance between greenhouse gas (GHG) emissions by anthropogenic sources and removals by sinks in the second half of the century^1^. Rapid promotion of the reduction of GHG emissions and increased removal of GHGs worldwide are important issues to realize the above goals.

**Figure 1:**
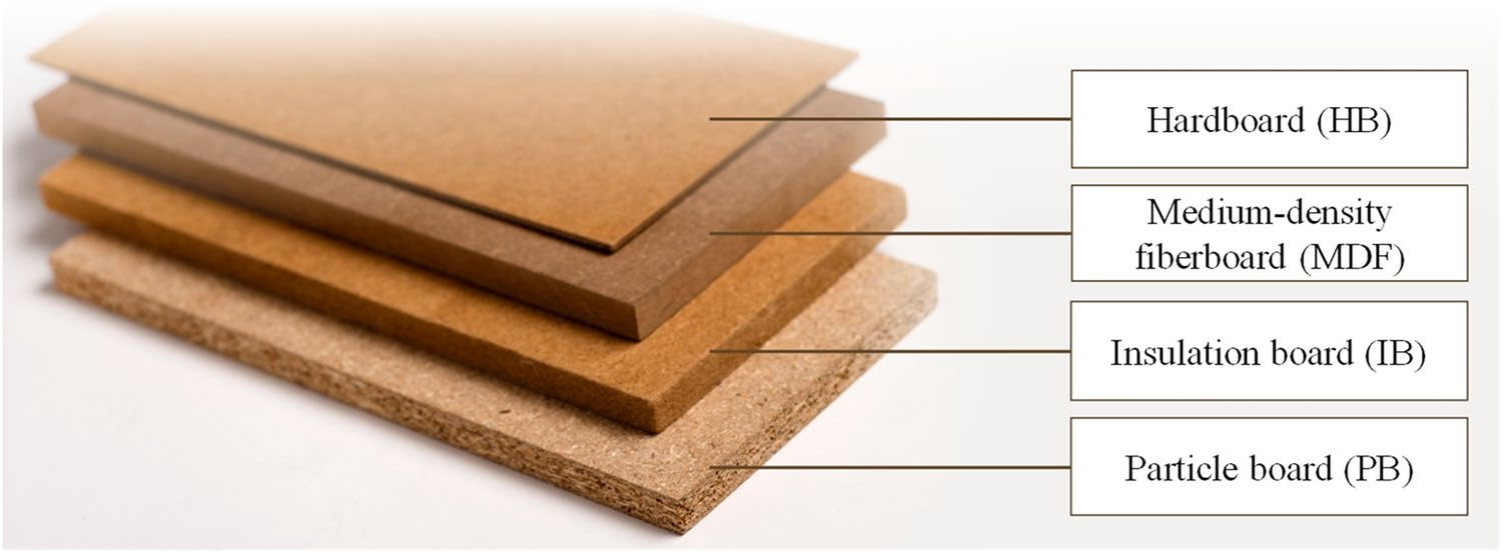
Particle board and fiberboard (i.e., hardboard, medium-density fiberboard, and insulation board)^8^.

Harvested wood products (HWPs) continue to store carbon even after they are harvested from forests; therefore, they affect the global carbon budget and are attracting attention as climate change countermeasures^2–7^. Among HWPs, particle board (PB) and fiberboard (FB) (Fig.1)^8^ usage is increasing on a global scale in recent years, and their global consumption has tripled over the past 30 years to approximately 268 million m^3^/year in 2021^9^. PB and FB prolong the carbon stock of HWPs through recycling, since many of these PBs and FBs are made from waste wood after use^10,11^. Therefore, it is important to quantitatively determine the amount of carbon stocks in PB and FB and their changes. Previous research estimated the carbon stock of HWPs as a whole, approximately including PB and FB^12–18^; however, there are no studies that focus on PB and FB in detail.

Japan has the largest HWP carbon stock after the United States, China, and Russia^6^. More than 90% of the raw materials for PB and FB were derived from forestry and wood processing residues and waste wood in Japan in 2021^19^, which is essential in the cascading use of wood. In particular, waste wood such as demolished building materials accounted for 71% of raw materials in 2021^19^; this contributes to prolonging the carbon storage period of HWPs. However, previous research on HWPs in Japan has not quantitated the amount of carbon stock by PB and FB^20–22^.

The purpose of this study was to estimate the carbon stocks of PB and FB in Japan from the past to present. Multiple methods for estimating the amount of carbon stock in HWPs have been presented by the Intergovernmental Panel on Climate Change (IPCC) guidelines^23–25^; however, the estimation results differ depending on the adopted method^26^. Therefore, this study used multiple estimation methods. Several HWP accounting approaches have been proposed, including the stock-change, production, atmospheric-flow, simple-decay approaches^23,25^. However, based on primary importance, we used the stock-change approach for wood products utilized to understand the PB and FB carbon stocks in Japan.

## Results

**Figure 2:**
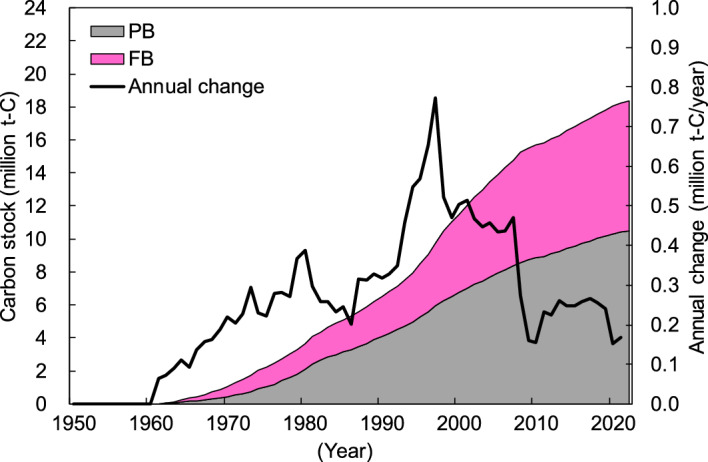
Japan’s particle board (PB) and fiberboard (FB) carbon stocks between 1961 and 2022 based on Tier 1 of the IPCC guidelines. “PB” includes FAOSTAT’s “Particle board and OSB (1961–1994),” “Particle board,” and “OSB”. “FB” includes “Fibreboard, compressed (1961–1994),” “Hardboard,” “MDF/HDF,” and “Other fibreboard”. “Annual change” represents the amount of annual change in carbon stock.

Japan’s estimated PB and FB carbon stocks based on the Tier 1 method of the latest 2019 IPCC guidelines^25^ between 1961 and 2022 are shown in Fig. 2. For Tier 1, first order decay (FOD) was used as the decay function, 25 years was used for the half-life, and the Food and Agriculture Organization (FAO) of the United Nations database (FAOSTAT)^9^ was used as the activity data for PB/FB consumption (see Methods section). Carbon stocks have continued to increase since 1961 and are estimated to reach a maximum of approximately 18.37 million t-C in early 2022. PB and FB accounted for approximately 10.51 million t-C (~ 57% of the total) and 7.86 million t-C (~ 43% of the total), respectively in 2022. The United Nations Framework Convention on Climate Change (UNFCCC) considers annual increases in carbon stocks as carbon removals and annual decreases as carbon emissions; therefore, the index of this annual change is also important. The annual change in carbon stock is positive throughout the target period (there are annual increases), and this annual increase continued until 1997, where it reached a maximum of approximately 0.77 million t-C/year. However, the subsequent annual increase has declined and the latest estimate for 2021 is approximately 0.17 million t-C/year.

**Figure 3:**
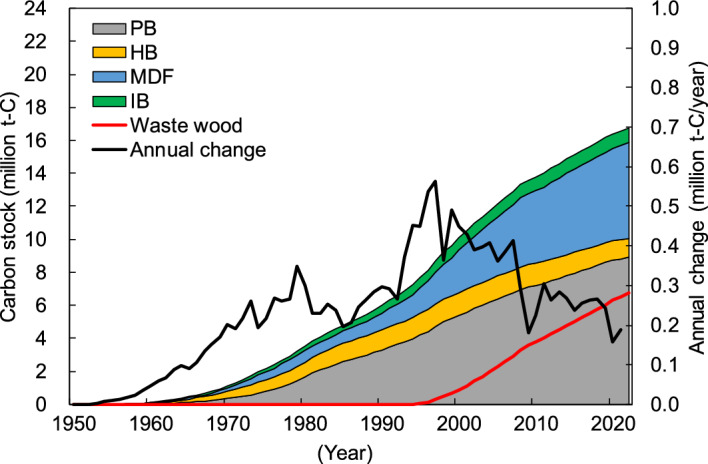
Japan’s particle board (PB) and fiberboard (FB) carbon stocks between 1953 and 2022 based on Tier 2 of the IPCC guidelines. HB: hardboard; MDF: medium-density fiberboard; and IB: insulation board. “Waste wood” refers to the amount of carbon stock derived from waste wood such as demolished building materials. “Annual change” represents the amount of annual change in carbon stock.

Japan’s estimated PB and FB carbon stocks based on the Tier 2 method of the IPCC guidelines^25^ between 1953 and 2022 are shown in Fig. 3. FOD was used as the decay function and 25 years was used for the half-life for Tier 2 using Japan-specific statistics^27^ for PB/FB consumption (see Methods section). Carbon stocks have continually increased since 1953 and are estimated to reach approximately 16.75 million t-C in early 2022. The breakdown was 53% for PB and 47% for FB (of which, 7% was hardboard (HB), 35% was medium-density fiberboard (MDF), and 5% was insulation board (IB)). Additionally, the carbon stock of PB and FB that used waste wood as the raw materials is estimated to be 6.77 million t-C in early 2022. This accounts for approximately 40% of the total carbon stock. The annual increase in carbon stock increased until 1997, reaching a maximum of approximately 0.56 million t-C/year, and then followed a decreasing trend. The latest estimate for 2021 is approximately 0.19 million t-C/year.

**Figure 4:**
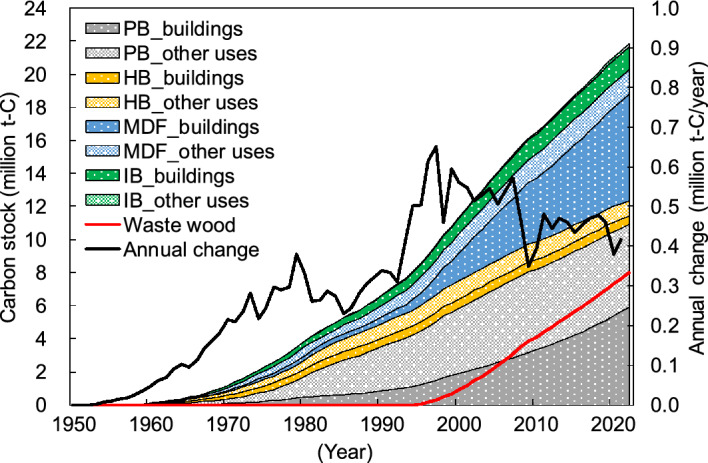
Japan’s particle board (PB) and fiberboard (FB) carbon stocks between 1953 and 2022 based on Tier 3 of the IPCC guidelines. HB: hardboard; MDF: medium-density fiberboard; IB: insulation board; “buildings” refers to building applications, and “other uses” refers to applications other than those used for buildings. “Waste wood” refers to the amount of carbon stock derived from waste wood such as demolished building materials. “Annual change” represents the amount of annual change in carbon stock.

Japan’s estimated PB and FB carbon stocks based on the Tier 3 method of the IPCC guidelines^25^ between 1953 and 2022 are shown in Fig. 4. For Tier 3, building PBs and FBs used a log-normal distribution as the decay function and a half-life of 38–63 years, and other PBs and FBs used FOD for the decay function and a half-life of 25 years; both used Japan-specific statistics^27^ as the data for PB/FB consumption (see Methods section). The carbon stock tended to increase more than that of Tier 1 and Tier 2 and was approximately 21.83 million t-C in early 2022. The breakdown was 50% PB and 50% FB (7% HB, 36% MDF, and 7% IB); 65% of the carbon stock was used for buildings and 35% was used for other uses. Additionally, the carbon stock of PB and FB that used waste wood as raw materials is estimated to be 7.99 million t-C in early 2022; this accounted for approximately 37% of the total carbon stock. The annual increase in carbon stocks peaked at approximately 0.65 million t-C/year in 1997, followed by a downward trend, with the latest estimate at approximately 0.42 million t-C/year for 2021.

## Discussion

There were similar trends of changes in carbon stocks over time for Tiers 1–3, and these trends reflected the socioeconomic conditions of Japan. Japan achieved rapid economic growth from the 1950s to 1970s^28^; therefore, the amount of carbon stock considerably increased (Figs. 2, 3 and 4), together with the consumption of PB and FB (Figs. 6 and 7). Additionally, the bubble economy increased the amount of carbon stocks from the late 1980s to the early 1990s^28^. However, the economic stagnation after the collapse of the bubble economy^28^ and the consumption tax hike in 1997^29^ resulted in peak PB and FB consumption in 1997, followed by a decrease and stagnation of the annual change of carbon stock. In particular, PB and FB consumption considerably decreased in 2008–2010 due to the impact of the global recession that originated in the United States, and the annual change in carbon stock showed a similar trend. Additionally, the large decrease in the annual variation of carbon stocks in 2020 is thought to be the influence of economic stagnation caused by the coronavirus 2019 pandemic.

**Figure 5:**
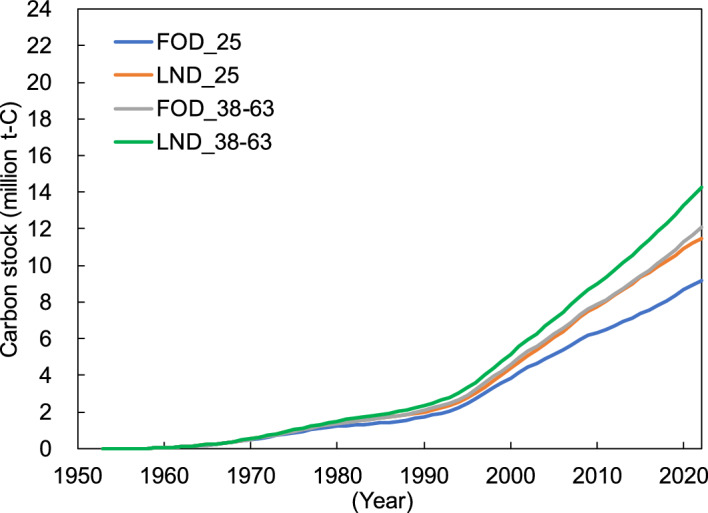
Japan’s building particle board (PB) and fiberboard (FB) carbon stocks between 1953 and 2022 under the different half-lives and decay functions. FOD_25: first order decay with a half-life of 25 years; LND_25: log-normal distribution with a half-life of 25 years; FOD_38–63: first order decay with a half-life of 38–63 years; LND_38–63: log-normal distribution with a half-life of 38–63 years. Values for “FOD_25” correspond to the building PB and FB carbon stocks based on Tier 2 shown in Fig. 3 while values for “LND_38–63” consist of the building PB and FB carbon stocks based on Tier 3 shown in Fig. 4.

Tier 1 carbon stocks are larger than those of Tier 2 (Figs. 2 and 3), and Tier 1 results were approximately 1.1 times of those of Tier 2 by early 2022. Meanwhile, the annual change in Tier 1 carbon stocks was approximately 0.9 times that of the Tier 2 results in 2021. Tier 1 and Tier 2 were estimated using the same decay function (FOD) and half-life (25 years) (see Methods section). Therefore, this difference is thought to be caused by the difference in consumption of PB and FB (the activity data). The FAOSTAT used in Tier 1 and country-specific statistics used in Tier 2 have slightly different definitions of PB and FB and may not cover common products. Additionally, PB and FB are often understood in units of area; therefore, the conversion factor to volume may differ. Furthermore, FAOSTAT includes FAO-specific estimates; therefore, it is unclear whether it reflects the actual circumstances of PB/FB consumption in Japan. Tier 1 is a useful method for countries and regions where country-specific statistics are insufficient and for international comparison studies using unified estimation methods and activity data. However, it is important to pay attention to the possibility that activity data from FAOSTAT do not agree with those from country-specific statistics which show an actual state of HWPs in the country.

Tier 3 carbon stocks are larger than Tier 2 carbon stocks, and Tier 3 results were approximately 1.3 times of Tier 2 results by early 2022 (Figs. 3 and 4). The annual change of Tier 3 carbon stock was approximately 2.2 times that of Tier 2 carbon stock. Tier 2 and Tier 3 use the same PB/FB consumption data. Meanwhile, Tier 2 uses FOD for the decay function and 25 years for the half-life, whereas Tier 3 uses the log-normal distribution for the decay function and 38–63 years for the half-life in the case of building PB and FB. Therefore, the difference between Tier 2 and Tier 3 results is owing to the difference in the decay functions and half-lives of building PBs and FBs.

We separately analyzed the effect of half-life and decay function on the difference between the results of Tiers 2 and 3. Japan’s estimated carbon stocks in building PB and FB under the different half-lives and decay functions are shown in Fig. 5. The difference in estimated results following changing decay functions of building PB and FB from FOD to log-normal distribution (the difference between “FOD_25” and “LND_25” in Fig. 5) was nearly equal to that observed by prolonging the half-lives of building PB and FB from 25 years to 38–63 years (the difference between “FOD_25” and “FOD_38–63” in Fig. 5). Therefore, it is thought that the choice of a decay function and a half-life almost equally affects the difference in estimated results between Tiers 2 and 3. The default half-life of 25 years in the IPCC guidelines^25^ is too short for the building PBs and FBs in Japan^30,31^. The amount of building PB and FB carbon stock from Tier 3 results was approximately 1.5 times that of Tier 2 results in early 2022. This is owing to Tier 3, which uses the longer half-life and reflects the suitable decay function of Japanese buildings (Fig. 5).

Approximately 40% of the carbon stock in PB and FB was derived from waste wood in Tier 2 and Tier 3 in early 2022. In 2021, forest residue, wood processing residue, and waste wood (e.g., demolished construction materials) account for 98% and 80% of the raw materials for PB and FB production in Japan, respectively^19^, and there is progress in the effective use of recycled materials. Among these, the percentage of waste wood in raw materials has been increasing, and this accounts for 94% for PB and 31% for FB in 2021 (Fig. 7)^19^. Waste wood was previously utilized as wood products such as logs, lumber, plywood, and glued laminated timber for construction, furniture, and so on, and they were discarded after playing the role of a carbon stock for a certain period of time. Therefore, the recycling of PB and FB can extend the carbon stock, making it an important carbon pool.

Carbon stock amounts were the highest in Tier 3, followed by Tier 1, and Tier 2 in early 2022. The latest annual changes in carbon stocks were highest in Tier 3, followed by Tier 2, and Tier 1 in 2021. It is thought that the Tier 3 carbon stock amount and its annual change are larger than those of the other two tiers as a result of applying the decay function and half-life that reflect the lifespan of Japanese wooden buildings to building PB and FB. Tier 3 is thought to have the highest estimation accuracy among the three tiers. This suggests that Tier 1 and Tier 2 underestimate the carbon stock amount and its annual change.

Carbon stocks in HWPs under the stock-change approach were approximately 386 million t-C in early 2019 in previous research covering entire HWPs in Japan^6^. Our results of PB and FB were approximately 21 million t-C in the same year based on Tier 3: this corresponded to nearly 5% of the carbon stocks in total HWPs. Carbon stocks in HWPs used in Japan’s buildings were estimated at approximately 148 million t-C in early 2019^22^, while our results of Tier 3 building PB and FB were approximately 13 million t-C in early 2019. This indicated 9% of the carbon stocks in building HWPs. In contrast, carbon stocks in HWPs annually decreased by approximately 2 million t-C/year in 2018 in Japan^6^. Meanwhile, this study showed that PB and FB annually increased by approximately 0.5 million t-C/year in the same year based on Tier 3. This contributes to an annual increase of over 20%. Annual decreases in carbon stocks in building HWPs were estimated at approximately 0.8 million t-C/year in 2018^22^. In contrast, our results of annual increases in building PB and FB by approximately 0.5 million t-C/year in the same year contribute to a nearly 60% increase in the annual changes. It is difficult to simply compare our results with those previous studies^6,22^ because there is a difference in estimation methods between them. Nevertheless, the decreasing trend of annual changes in carbon stocks in HWPs is mitigated by the annual increase of PB and FB carbon stocks.

This study referred to the latest IPCC guidelines^25^ for the estimation method; the estimation methods in the 2006 guidelines^23^ and 2013 guidance^24^ are slightly different from those in the 2019 guidelines^25^ in terms of target wood products, half-lives, carbon conversion factors, and other aspects^6^. The results of carbon stock estimation will differ if other guidelines are referenced. The 2006 guidelines^23^ and the 2019 guidelines^25^ provide estimation methods under the stock-change approach. Half-lives and carbon conversion factors differ between the two guidelines with respect to PBs and FBs. Therefore, we analyzed the effect of a difference in IPCC guidelines on the estimated results of carbon stocks. We changed half-lives from 25 years (the 2019 guidelines)^25^ to 30 years (the 2006 guidelines)^23^ for PB/FB based on Tiers 1–2 and PB/FB for uses other than buildings based on Tier 3 and as carbon conversion factors from the values in Table 1 (the 2019 guidelines)^25^ to 0.294 t-C/m^3^ (the 2006 guidelines)^23^ for PB/FB based on Tiers 1–3. The estimated results of the carbon stocks are shown in Supplementary Figs. S1–S3. The carbon stocks and their annual changes were greater under the 2006 guidelines compared with those under the 2019 guidelines for all Tiers. The estimation using the latest guidelines^25^ in this study leads to conservative results compared to that in the previous guidelines^23^.

**Table 1:** Carbon conversion factors.

	t/m^3^	t-C/t	t-C/m^3^
Particle board	0.596	0.451	0.269
Hardboard	0.788	0.425	0.335
Medium-density fiberboard	0.691	0.427	0.295
Insulating board	0.159	0.474	0.075
Oriented strandboard	0.573	0.463	0.265
Fiberboard, compressed	0.739	0.426	0.315

A limitation of this research is that we were unable to examine the decay function and half-life for uses other than that for buildings. PB and FB are used in applications such as construction, furniture, electrical equipment, automobile interiors, packaging, and miscellaneous goods^32^. The FOD function and 25-year half-life suggested in the latest IPCC guidelines^25^ are likely to be different for these applications. However, the decay function and half-life that indicate the actual circumstances were not scientifically elucidated. Therefore, a default FOD of 25 years was applied in this study. It is thought that the estimation accuracy of PB and FB carbon stocks would be further improved by determining the suitable decay functions and half-lives for each application.

**Figure 6:**
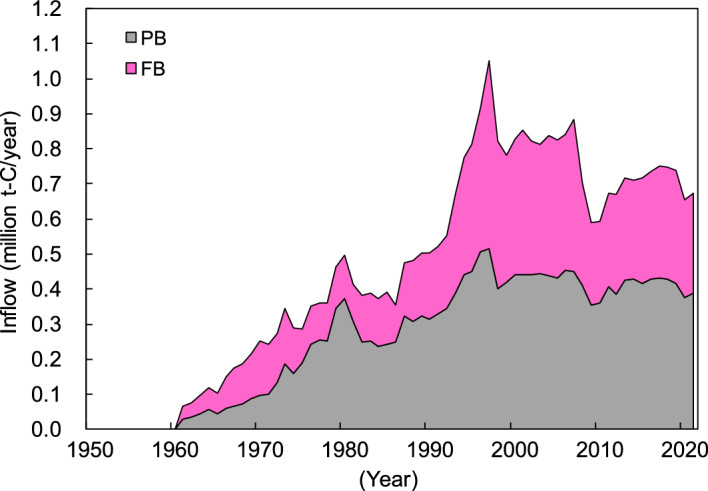
Japan’s consumption of particle board (PB) and fiberboard (FB) from 1961 to 2021 obtained from the Food and Agriculture Organization (FAO) of the United Nations database (FAOSTAT) (carbon equivalent).

**Figure 7:**
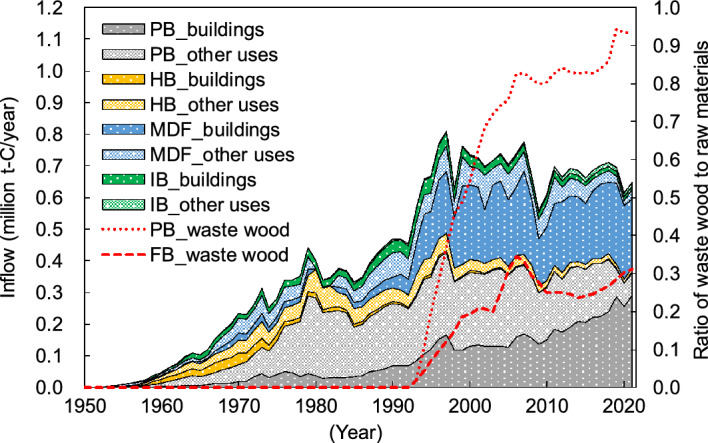
Japan’s consumption of particle board (PB) and fiberboard (FB) from 1953 to 2021 obtained from Japan-specific statistics (carbon equivalent). HB: hardboard; MDF: medium-density fiberboard; IB: insulation board; “buildings” refers to building applications, and “other uses” refers to applications other than for buildings. “Waste wood” refers to the ratio of waste wood (such as demolished building materials) to raw PB and FB materials.

Another limitation of this study is that it only targets the stock-change approach as an accounting approach for HWPs. Under the Paris Agreement, both the Nationally Determined Contributions and National Greenhouse Gas Inventory Report (NIR) allow countries to freely choose their HWP accounting approach^33,34^. However, the NIR is required to report production approach figures as supplementary information^34^. The carbon stock amount and its annual change will greatly vary depending on the accounting approach^2,3,6,13^. Therefore, it is important to consider accounting approaches other than the stock-change approach.

## Conclusions

This study estimated the amount of PB and FB carbon stocks and their annual changes over the past 70 years (1953–2022) in Japan. Each value was estimated using three methods indicated in the latest IPCC guidelines.

Japan’s PB and FB carbon stocks continually increased using Tier 1–3 methods, although the annual increase in carbon stocks peaked in 1997, followed by a downward trend. The carbon stock amount and the amount of annual change were larger in Tier 3 compared with Tier 1 and Tier 2. This was influenced by the use of decay functions and half-lives for building PB/FB that are suitable for Japanese wooden buildings. Therefore, it was suggested that Tier 3 has the highest estimation accuracy, whereas Tiers 1 and 2 underestimate carbon stocks and annual changes. Approximately 40% of the PB and FB carbon stock is derived from waste wood, which extends the carbon stock lifetime. While contribution of carbon stocks in PB and FB to those in all HWPs is limited, their annual increases greatly contribute to mitigate decreasing trends of annual changes in carbon stocks in HWPs. Therefore, further effective use of PB and FB strongly promotes carbon removal of HWPs in Japan.

This study provides numerical information of the amount of changes in carbon stock in PBs and FBs over time which plays an important role in the cascading use of wood resources for policy makers, experts, and researchers that are involved in wood utilization policies and climate change countermeasures. The findings will contribute to the examination of climate change countermeasures through the effective use of PBs and FBs. Additionally, the methods used in this research can be applied to countries and regions other than Japan, and to HWPs other than PBs and FBs.

## Methods

### Target products and period

This research focused on the PBs and FBs used in Japan from the entire available statistical data between 1953 and 2022 to elucidate the amount of carbon stock.

### Carbon stock amount estimation methods

The latest IPCC guidelines present three methods for estimating the carbon stock amount and its annual changes (Tiers 1–3) depending on the methods applicable in each country and the availability of HWP activity data^25^. The estimation results differ depending on the tier^25,26^; therefore, these three methods were used to estimate carbon stock amounts in this study.

#### Tier 1

Tier 1 is an estimation method that is applied when country-specific methods cannot be applied, and country-specific HWP activity data cannot be used. This method applies FOD, which is a default decay function that is proposed by the IPCC guidelines^25^. The activity data used FAOSTAT^9^, the global forest product statistics published by FAO. Eqs. (1)–(3) show the estimation formulas for the carbon stock amount and its annual changes using FOD:

1 $$C\left( {i + 1} \right) = e^{ - k} \cdot C\left( i \right) + \frac{{1 - e^{ - k} }}{k} \cdot Inflow\left( i \right)$$

2 $$k = \frac{{{\text{ln}}\left( 2 \right)}}{HL}$$

3 $$\Delta C\left( i \right) = C\left( {i + 1} \right) - C\left( i \right)$$

where $C\left(i+1\right)$ is the PB and FB carbon stock at the beginning of year $i+1$ (t-C), $Inflow\left(i\right)$ is the carbon input amounts of PB and FB into the carbon stock during year $i$ (t-C/year), $HL$ is the half-life of PB and FB (year), $\Delta C\left(i\right)$ is the annual change of carbon stock during year $i$ (t-C/year), and $i$ indicates each year from 1961 to 2021.

$Inflow\left(i\right)$ uses FAOSTAT data. These international statistics are available from 1961 onwards. The classification of PB and FB commodities changed between pre-1994 and post-1995 (Supplementary Fig. S4). The “Particle board” and “OSB” (oriented strandboard) of FAOSTAT were targeted for PB. Particle board and OSB commodities were combined prior to 1994 and were only separated from 1995 onwards. Meanwhile, for FB, prior to 1994, “Hardboard” and “MDF/HDF” were combined as “Fibreboard, compressed (1961–1994)”, and since 1995, these commodities have been separated. Additionally, “Other fibreboard” was set as IB^35^. Production, import, and export data (m^3^/year) of these commodities were obtained, and the consumption (= production + import – export) (m^3^/year) was calculated. This was multiplied by the carbon conversion factor (t-C/m^3^)^25^ of the IPCC guidelines shown in Table 1 to convert it to a carbon amount (t-C/year), which was then used as the amount of input to carbon stock as shown in Fig. 6.

The $HL$ was set to 25 years^25^, which was indicated as a default value for wood-based panels in the IPCC guidelines.

#### Tier 2

Tier 2 is a method that uses country-specific activity data using FOD as the decay function. The FOD function is the same as that for Tier 1 Eqs. (1)–(3).

PB and FB consumption data were used as activity data based on Japan-specific statistics^27^ for the $Inflow\left(i\right)$ in Eq. (1). Specifically, domestic consumption (= domestic sales + import) data were obtained for PB and FB (HB, MDF, and IB) by application from 1953 to 2021 from the Japan Fiberboard and Particleboard Manufacturers Association (JFPMA). Supplementary Data show the numerical data of the statistics. This PB and FB consumption amount (m^3^/year, t/year) was multiplied by the carbon conversion factor (t-C/m^3^, t-C/t) (Table 1) to convert it to a carbon amount (t-C/year), which was then used as the amount of input to carbon stock (Fig. 7). Products corresponding to “OSB” in FAOSTAT^9^ of Tier 1 are included in the PB of Japan-specific statistics^27^.

Time-series data of the breakdown of raw materials were also obtained for PB and FB from JFPMA^27^. The PB and FB consumption that was derived from waste wood was calculated by multiplying the PB and FB consumption by the ratio of waste wood (e.g., demolished construction materials) to raw PB and FB materials for each year. Japan-specific statistics for the ratio of waste wood data were only available for the following periods: 1993 and 1998–2021^27^. Therefore, data between 1994 and 1997 were interpolated by linearly connecting the two ratios in 1993 and 1998, and the ratios between 1953 and 1992 were assumed to be 0 (see Supplementary Data). Subsequently, the PB and FB consumption that was derived from waste wood was substituted into $Inflow\left(i\right)$ in Eq. (1), and the amount of PB/FB carbon stock derived from waste wood was estimated.

A uniform 25 years $HL$^25^ was set for buildings and other uses; this was the same as that used for Tier 1.

#### Tier 3

Tier 3 is a method that uses a country-specific estimation method and activity data. Japanese wooden buildings using PB and FB have reported half-lives of 38–63 years^30^, which is much longer than the default value of 25 years in the IPCC guidelines^25^. The log-normal distribution is the best fit for the decay function, not the FOD^30^. It is thought that taking these factors into account will result in a higher estimation accuracy of PB and FB carbon stocks than using FOD as the decay function and 25 years as the half-life; therefore, we applied country-specific estimation methods for building PBs and FBs. Eqs. (4) and (5) show the carbon stock estimation formulas using the log-normal distribution as the decay function:

4 $$Cb\left( {i + 1} \right) = \mathop \sum \limits_{{n = i_{0} }}^{i} \left[ {Inflowb\left( n \right) \cdot R\left( {i - n} \right)} \right]$$

5 $$R\left( {i - n} \right) = 1 - \frac{1}{{\sqrt {2\pi } }}\mathop \smallint \limits_{0}^{i - n} \frac{1}{x}exp\left[ { - \frac{{\left( {ln\,x - \mu } \right)^{2} }}{{\sigma^{2} }}} \right]dx$$

where $Cb\left(i+1\right)$ is the building PB and FB carbon stock amount at the beginning of year $i+1$ (t-C), $Inflowb\left(n\right)$ is the input amount of building PB and FB into carbon stock during year $n$ (t-C/year), and $R\left(i-n\right)$ is the remaining fraction of the PB and FB input to carbon stock after $i-n$ years. Half-life ($HL$) is defined as the number of years elapsed ($i-n$) in which the remaining fraction attains 0.5. $\mu$ refers to the mean and signifies the natural logarithm of the half-life ($HL$), $\sigma$ refers to the standard deviation, ${i}_{0}$ refers to the initial year of 1953, and $i$ refers to each year up to 2021.

$Inflowb\left(n\right)$ targets those for building applications among Tier 2 country-specific statistical data (Fig. 7). The import data were unavailable by application, whereas the domestic sales data were available. Therefore, we determined the input amount of imported building PB and FB via the ratio of building PB and FB in the domestic sales data for each year (see Supplementary Data).$i$=1953–1964 had a half-life of 38 years and standard deviation of 0.60, $i$=1965–1996 had a half-life of 56 years and standard deviation of 0.61, and $i$=1997–2021 had a half-life of 63 years and standard deviation of 0.20 based on previous research estimating the lifetime distribution of wooden buildings^30^.

The amount of carbon stock derived from waste wood was also estimated using the ratio of waste wood in the raw PB and FB materials each year, as in Tier 2.

The decay function and half-life of PBs and FBs for uses other than for buildings are not clarified; therefore, we used FOD for the decay function and 25 years for the half-life, as in Tier 2.

## Supplementary Information

Supplementary Information 1.

Supplementary Figures.

## Data Availability

The datasets generated and analyzed during the current study are available from the corresponding author on reasonable request.
