# Supplementary material for: Carbon stocks of particle board and fiberboard in Japan
Source: Sci Rep. 2023 Jun 17;13:9846. doi: 10.1038/s41598-023-37132-x (PMC10276831; doi:10.1038/s41598-023-37132-x)
Supplement: Supplementary file 2 — Supplementary Figures. [file 41598_2023_37132_MOESM2_ESM.docx]

**Supplementary Figures**

**Figure S1.** Japan’s particle board (PB) and fiberboard (FB) carbon stocks between 1961 and 2022 based on Tier 1 of the 2006 IPCC guidelines^[1]^. *Note*: “annual change” represents the amount of annual change in carbon stock. This figure shows the estimated results that prolonged the half-lives to 30 years and changed the carbon conversion factors to 0.294 t-C/m^3^ of PB and FB following the 2006 guidelines^[1]^ from the results in Fig. 2.

**Figure S2.** Japan’s particle board (PB) and fiberboard (FB) carbon stocks between 1953 and 2022 based on Tier 2 of the 2006 IPCC guidelines^[1]^. *Note*: HB: hardboard; MDF: medium-density fiberboard; and IB: insulation board. “Waste wood” refers to the amount of carbon stock derived from waste wood such as demolished building materials. “Annual change” represents the amount of annual change in carbon stock. This figure shows the estimated results that prolonged the half-lives to 30 years and changed the carbon conversion factors to 0.294 t-C/m^3^ of PB and FB following the 2006 guidelines^[1]^ from the results in Fig. 3.

**Figure S3.** Japan’s particle board (PB) and fiberboard (FB) carbon stocks between 1953 and 2022 based on Tier 3 of the 2006 IPCC guidelines^[1]^. *Note*: HB: hardboard; MDF: medium-density fiberboard; IB: insulation board; “buildings” refers to building applications, and “other uses” refers to applications other than those used for buildings. “Waste wood” refers to the amount of carbon stock derived from waste wood such as demolished building materials. “Annual change” represents the amount of annual change in carbon stock. This figure shows the estimated results that prolonged the half-lives to 30 years and changed the carbon conversion factors to 0.294 t-C/m^3^ of PB and FB for other uses following the 2006 guidelines^[1]^ from the results in Fig. 4.

**Figure S4.** Shifts in particle board (PB) and fiberboard (FB) commodity classification in the Food and Agriculture Organization (FAO) of the United Nations database (FAOSTAT) ^[2]^.

1. Intergovernmental Panel on Climate Change (IPCC). IPCC Guidelines for National Greenhouse Gas Inventories. 2006 (National Greenhouse Gas Inventories Programme. Inst. for Global Environmental Strategies, Hayama, Japan, 2006).
2. Food and Agriculture Organization of the United Nations (FAO). FAOSTAT. Forestry production and trade. https://www.fao.org/faostat/en/#data/FO (2023).
